# How to measure distance visual acuity

**Published:** 2019-12-17

**Authors:** Janet Marsden, Sue Stevens, Anne Ebri

**Affiliations:** 1 Former Nursing Advisor: *Community Eye Health Journal*, International Centre for Eye Health, London School of Hygiene & Tropical Medicine, UK.; 2 Former Nursing Advisor: *Community Eye Health Journal*, International Centre for Eye Health, London School of Hygiene & Tropical Medicine, UK.; 3 West Africa Sub-Regional Manager: Brien Holden Vision Institute, Calabar, Nigeria.

**Visual acuity is a measure of the ability of the eye to distinguish the details of objects.**

**Figure:**
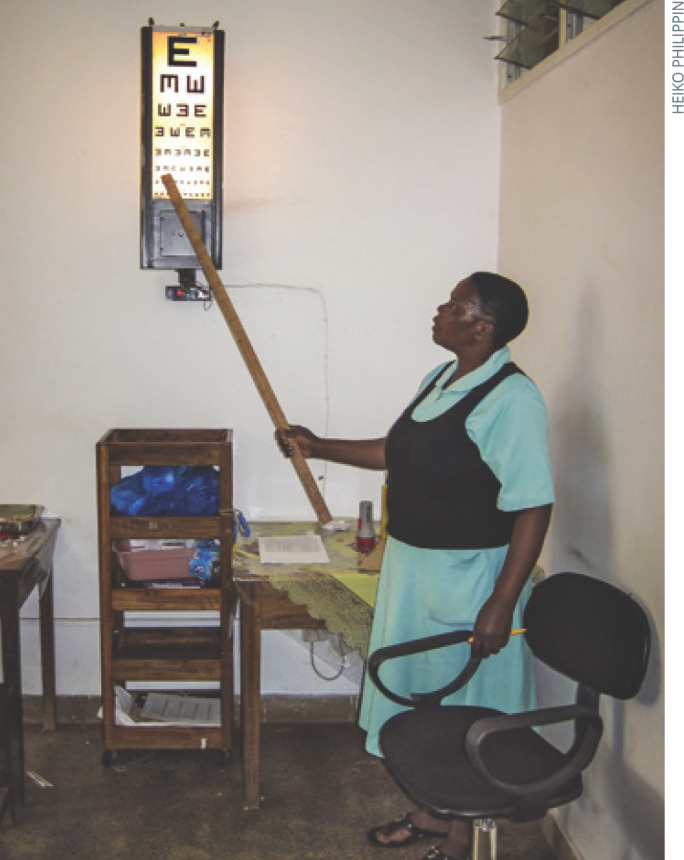
Visual acuity should be measured from a standard distance, using a standard chart. TANZANIA

Visual acuity testing is part of every eye examination. It is important that it is done well, and accurately, as an incorrect measurement can lead to inappropriate decisions and management.

It is important to assess visual acuity in a consistent way in order to detect any changes in vision. One eye is tested at a time.

## Equipment

- Multi-letter Snellen chart or tumbling E (or C) chart
- Plain occluder, card or tissue
- Pinhole occluder
- Patient's documentation

## Preparation

- Ensure good natural light or illumination on the chart.
- Explain the test to the patient.
- Tell the patient it is not a test that they have to pass. Tell them not to guess if they cannot see.
- Position the patient, sitting or standing, six metres away from the 6-metre Snellen or tumbling E chart (or 3 metres away from the 3-metre Snellen or E chart).

## Testing and recording visual acuity

- Test the eyes one at a time, usually starting with the right eye, without any spectacles.
- Ask the patient to cover the left eye with the plain occluder, card or tissue.
- Ask the patient to read from the top of the chart and from left to right. For children or adults who cannot read the letters, use a tumbling E or C chart and ask them to point in the direction that the ‘legs’ of the E (or the opening in the C) are facing. There is a one in four chance that the patient can guess the direction; the patient should therefore correctly indicate the orientation of **most** letters of the same size, e.g., three out of four.
- Record the visual acuity for the examined eye. Visual acuity is expressed as a fraction e.g. 6/18. The top number is the distance the patient is from the chart in metres (6). The bottom number is the **smallest** line on the chart the person can read accurately. For example the 18 line (6/18), or the 6 line (6/6).
- Incomplete lines can be added to the last complete line. e.g. 6/12+3, indicating that the patient read the ‘12’ line at 6 metres and three of the letters on the ‘9’ line.

**The pinhole test**

Using a pinhole reduces the need to focus the light that enters the eye, and people with a refractive error, such as myopia, can usually see better with the pinhole than without it.

**Steps**

- Position the patient 6 metres from the chart.
- Ask the patient to cover one eye with the occluder.
- Position the pin hole over the eye to be tested so they can see the chart through the pinhole.
- Test one eye at a time by following the same procedure used to test visual acuity.

If the person can read more letters with the pinhole than without, they are likely to have a refractive error, such as myopia. All patients (adults and children) whose acuity improves with a pinhole should undergo a full refraction to see whether they require spectacles, and of what power.

If the patient cannot read the largest (top) letter at 6 metres, either:

– move them closer to the chart, 1 metre at a time, until the top letter can be seen – the VA will then be recorded as 5/60 or 4/60, etc. or
– hold up your fingers at varying distances (5 metres, 4 metres etc. and record the vision as counting fingers (CF) at the maximum distance they can see between 5 and 1 metre, i.e. **VA = CF 5m or VA = CF 1m**.

If the patient cannot count fingers at 1 metre, wave your hand and check if he/she can see this. This is recorded as hand movements (HM): **VA = HM**.

If the patient cannot see hand movements, shine a torch in the eye and ask if they can see the light. If they can, record ‘perception of light’: **VA = PL**. If they cannot see the light, record ‘no perception of light’: **VA = NPL**.

- After testing and recording the VA for the right eye repeat now for the left eye.
- If the patient wears spectacles for distance vision, now test the VA in each eye with the spectacles on.
- If the visual acuity in either eye is less than 6/6, one can measure the visual acuity with a pin hole (see panel).
- The VA is recorded for each eye in the patient's notes. For example:
  Right VA = 6/18 without spectacles
  Right VA = 6/6 with spectacles
  Left VA = HM without spectacles
  Left VA = HM with spectacles
